# Computational Investigation to Design Ofloxacin-Loaded Hybridized Nanocellulose/Lipid Nanogels for Accelerated Skin Repair

**DOI:** 10.3390/gels8090593

**Published:** 2022-09-16

**Authors:** Mona M. AbouSamra, Nada M. El Hoffy, Nahla A. El-Wakil, Ghada E. A. Awad, Rabab Kamel

**Affiliations:** 1 Pharmaceutical Technology Department, National Research Centre, Giza 12622, Egypt; 2 Faculty of Pharmacy, Future University in Egypt, New Cairo 11835, Egypt; 3 Cellulose and Paper Department, National Research Centre, Giza 12622, Egypt; 4 Chemistry of Natural and Microbial Product Department, National Research Centre, Giza 12622, Egypt

**Keywords:** lipid, nanocellulose, nanogels, factorial, regression, anti-bacterial assay, wound healing, cytocompatibility

## Abstract

The pharmaceutical application of biomaterials has attained a great success. Rapid wound healing is an important goal for many researchers. Hence, this work deals with the development of nanocellulose crystals/lipid nanogels loaded with ofloxacin (OFX) to promote skin repair while inhibiting bacterial infection. Ofloxacin-loaded hybridized nanocellulose/lipid nanogels (OFX-HNCNs) were prepared and evaluated adopting a computational method based on regression analysis. The optimized nanogels (OFX-HNCN7) showed a spherical outline with an encapsulation efficiency (EE), particle size (PS) and zeta potential (ZP) values of 97.53 ± 1.56%, 200.2 ± 6.74 nm and −26.4 ± 0.50 mV, respectively, with an extended drug release profile. DSC examination of OFX-HNCN7 proved the amorphization of the encapsulated drug into the prepared OFX-HNCNs. Microbiological studies showed the prolonged inhibition of bacterial growth by OFX-HNCN7 compared to the free drug. The cytocompatibility of OFX-HNCN7 was proved by Sulforhodamine B assay. Tissue repair was evaluated using the epidermal scratch assay based on cell migration in human skin fibroblast cell line, and the results depicted that cell treated with OFX-HNCN7 showed a faster and more efficient healing compared to the control. In overall, the obtained findings emphasize the benefits of using the eco-friendly bioactive nanocellulose, hybridized with lipid, to prepare a nanocarrier for skin repair.

## 1. Introduction

Skin wounds can cause disability and even death in severe cases, so this is an important issue that requires attention [1,2]. Currently, chronic skin wounds remains a clinical challenge, with more than half of chronic wounds revealing resistance to conventional therapies [3,4]. Moreover, damaged tissues are vulnerable to bacterial infection which can pass to the subcutaneous layer before being transported by the bloodstream to various tissues, causing severe toxic effects [5]. In consequence, the direction towards a multi-purpose formulation is an essential demand.

Recently, the demand for bio-based materials that can enhance the wound healing process has grown significantly [6,7]. Biomaterials are commonly employed due to their compatibility with the extracellular matrix, which helps cells to multiply and differentiate efficiently while also facilitating the restoration of damaged tissues, speeding up the natural healing process [8]. Cellulose is the most commonly used biopolymer [3,9,10], it can be generated from agricultural waste using different treatment methods. The emulsion stabilizer properties of cellulose nanocrystals (NC) are attributed to its amphiphilic nature as it consists of a hydrophobic backbone and numerous hydroxyl groups; the adsorption of this biological macromolecule at the oil/water interface form a protective film around the emulsified droplets [11,12,13]. Due to the combined beneficial properties of nanocellulose like the ability to prolong drug release [14,15], stabilize emulsions [16,17], and enhance the regeneration of cells [13,15,17] besides its potential antimicrobial properties [18], it was used with great success in wound healing preparations [17,19,20,21]. On the other hand, lipid type nanocarriers have proven a great success as dermal preparations for protective as well as therapeutic purposes [22,23,24,25,26,27]. The study aimed to combine the beneficial effects of lipid nanocarriers and nanocellulose to prepare an optimized nanoformula for dermal repair.

As cited before, microbial contamination represents a key contest confronting wound healing, which urges the need for prophylactic antibiotic incorporation into wound dressings. Ofloxacin (OFX) is a fluoroquinolone antibiotic capable of fighting a wide range of bacterial infections [28]. Some studies proved the efficiency of Ofloxacin to combat bacterial infection which is the main cause of delayed wound healing [29,30,31,32]. However, the simultaneous combination between ofloxacin and cellulose nanocrystals has not been yet investigated.

The current study focuses on the feasibility of producing an antimicrobial wound healing preparation incorporating OFX-loaded hybridized nanocellulose/lipid nanogels (OFX-HNCNs). In order to accurately make predictions while saving cost, time and efforts; a computational method was used. The design and optimization of the nano-formulations were done based on a 2^3^ full-factorial experimental design after which a regression analysis was performed to study the effect of the chosen factors on the physicochemical properties of the produced nanogels to select the optimum formulation. Then, the optimized OFX-HNCN was subjected to characterization, microbiological and cell biology studies comprising cell viability and wound healing testing.

## 2. Results and Discussion

### 2.1. Characterization of the Ofloxacin-Loaded Hybridized NC/Lipid Nanogels

#### 2.1.1. Particle Size Analysis

The particle size analysis results are listed in Table 1; the particle size of all formulae was ranging between 173.5 ± 11.01 and 268.3 ± 11.32 nm, which seems to be suitable to assure dermal accumulation rather than penetration to the systemic circulation. It is well documented that a small particle size permits for transdermal permeation and allows for the drug to reach the systemic circulation [33]. At the same time, some previous studies have proved that nanogels with a size ranging from about 200 to 400 nm can accumulate in the skin allowing for a dermal site-specific effect and low systemic penetration [22,23,24,25]. ZP values were between −22.3 ± 1.01 and −29.6 ± 0.84 mV, which decreases the chance for particle aggregation [34]. It was previously reported that high ZP values assure the electrostatic repulsion between nanogels [35]. These results can indicate the efficacious emulsification helping in the formation of a system with fine well-separated droplets. It was previously reported that the combination of mixed emulsifiers of different nature could permit for a higher reduction in surface tension and better stabilization [22,23,34]. The formation of a mixed layer of the used emulsifiers (P188 and NC) at the interface can increase the emulsification efficacy resulting in the successful preparation of the hybridized NC/lipid nanogels. The negative ZP values can be attributed to the NC molecules bearing sulfate and hydroxyl groups [36].

The PDI values ranged from 0.4 ± 0.01 to 0.6 ± 0.02. The PDI is used to describe the degree of non-uniformity of size distribution of particles. It is generally known that, if the PDI is more than 0.7, the sample has a very broad distribution [37], while values less than 0.5 indicate the formation of monodisperse samples with a narrow distribution and a low tendency for aggregation [38].

The best fitting equation presenting the particle size (PS) is:

PS = 226.25 + 8.15X1 + 7.88X2 − 1.73X3 − 1.03X1X2 − 0.38X1X3 + 4.85X2X3

(R^2^ = 0.9998, F = 773.08, *p* < 0.05)

According to Table 2, after excluding the insignificant terms, the reduced model polynomial equation is a following:

PS = 226.25 + 8.15X1 + 7.88X2 − 31.73X3 + 4.85X2X3

**Figure 1:**
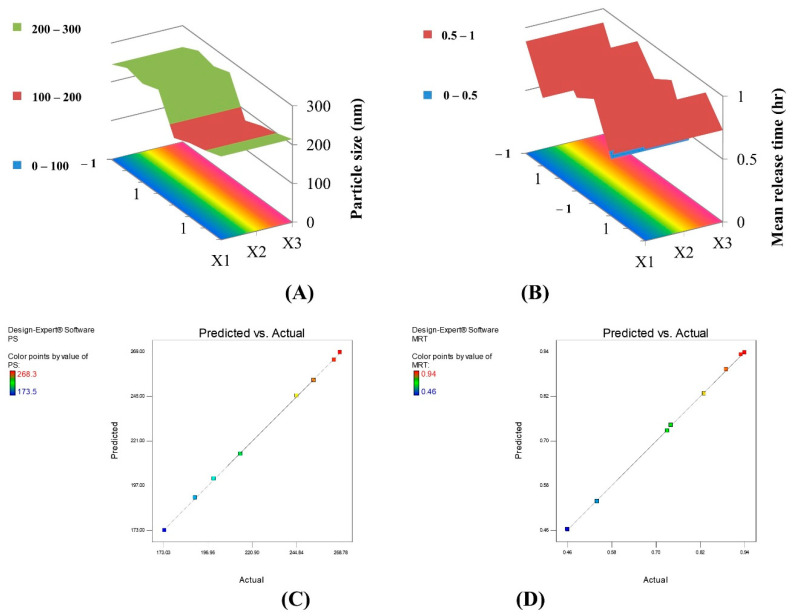
(**A**) The 3D surface response plot representing Y1 (PS). (**B**) The 3D surface response plot representing Y5 (MRT). (**C**) Predicted versus actual plot (PS). (**D**) Predicted versus actual plot (MRT).

The adopted model demonstrated good agreement between the values of the R^2^ (0.9998), adjusted R^2^ (0.9986), and predicted R^2^ (0.9876), as well as a precision value of 76.190, which ensures the model’s sufficiency and adequate signal, which confirms that the current model can be used to explore the entire design space. This is confirmed by the predicted versus actual graph presented in Figure 1C. Based on the equation, it can be detected that increasing the lipid and P188 concentrations increased the particle size; this can be attributed to the high melting point of the lipid (GMS), which results in slow crystallization after the hot homogenization condition ensuring an increase in the particle size. This can also be due to the more difficult emulsification by the increment of the lipophilic component. Whereas, increasing the concentration of NC significantly decreased the PS, which can be explained by the emulsifying effect of NC molecules, a small amount of NC may not be sufficient to cover the oil/water interface resulting in the aggregation of lipid droplets leading to larger particle size. Previous reports indicated that NC could act as an emulsion stabilizer due to its amphiphilic structure composed from the hydrophobic crystalline backbone with numerous hydrophilic hydroxyl groups; the adsorption of these solid nanogels between the oil/water interface form a protective layer surrounding the emulsified droplets [11,12,13]. A previous study assigned that NC molecules arrange themselves as a monolayer along the crystal plane (2 0 0), which prohibits the deformation of the oily interface [39].

The combination between X2 and X3 had a synergistic effect.

The 3D surface response plot representing Y1 is shown in Figure 1A.

The best fitting equation presenting the polydispersity index (PDI) is:

PDI = 0.53 + 0.028X1 + 0.022X2 + 0.007X3 + 0.065X1X2 + 0.025X1X3 − 0.045X2X3

(R^2^ = 0.86, F = 0.50, *p* > 0.1)

This model proved to be insignificant; thus Y2 (PDI) was not included in the optimization study.

The best fitting equation presenting the ZP is:

ZP = −26.59 + 0.46X1 + 0.96X2 −1.01X3 + 0.76X1X2−0.91X1X3 + 0.19X2X3

(R^2^ = 0.98, F = 4.58, *p* > 0.1)

This model proved to be insignificant; thus Y3 (ZP) was not included the optimization study.

#### 2.1.2. Encapsulation Efficiency (EE%)

The values of the EE are listed in Table 1; all formulae had high values (above 95%) which prove the efficient emulsification.

The best fitting equation presenting the encapsulation efficiency (EE) is:

EE = 97.02 + 0.0001X1 + 0.39X2 + 0.22X3 − 0.21X1X2 − 0.04X1X3 + 0.05X2X3

(R^2^ = 0.77, F = 0.24, *p* > 0.1)

This model proved to be insignificant; thus Y4 (EE) was not included in the optimization study.

#### 2.1.3. In Vitro Release Study

**Figure 2:**
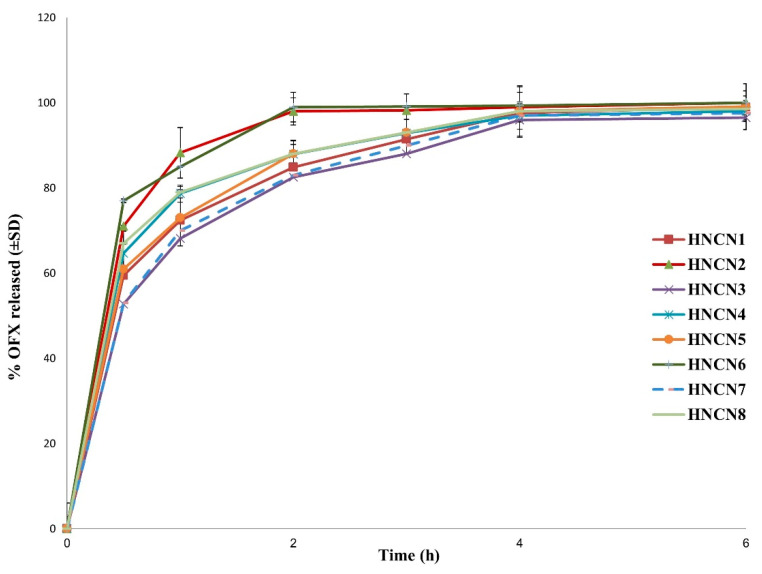
In-vitro OFX release profiles from different ofloxacin-loaded hybridized nanocellulose-lipid nanogels formulations (HNCNs) in phosphate buffer pH 7.4 at 37 °C.

Figure 2 depicts the drug release pattern from the designed formulae. A biphasic pattern was seen in all HNCNs. The initial burst release (at 30 min) can be attributed to the instantaneous dissolution of the OFX that has been adsorbed on the nanogels’ surface, followed by a delayed drug release due to the progressive escape of the drug contained in the core. The initial quick drug release can be advantageous since it ensures a high drug concentration at the action site which would help to attain the therapeutic concentration in a minimal time. The retardation of drug release after the first hour may also be due to the capture of water molecules and swelling of nanocellulose by time, leading to the formation of a tortuous pathway making a long journey for the drug molecules to travel to the dissolution medium.

The best fitting equation presenting the mean release time (MRT) is:

MRT (Y5) = 0.76 − 0.14X1 + 0.08X2 − 0.02X3 + 0.04X1X2 − 0.001X1X3 + 0.01X2X3

(R^2^ = 0.9998, F = 125.96, *p* < 0.1)

According to Table 3, after exclusion of the insignificant terms, the reduced model polynomial equation will be:

MRT = 0.76 − 0.14X1 + 0.08X2 + 0.01X1X2

The adopted model showed excellent correlation between the values of the R^2^ (0.9998), adjusted R^2^ (0.9984), and predicted R^2^ (0.9856), in addition to a precision value of 71.813, which guarantees the model’s sufficiency and adequate signal, which confirms that the current model can be used to explore the entire design space as shown in Figure 1D which demonstrates the predicted versus actual values of MRT. Increasing lipid concentration decreased MRT; this can be due to the slow crystallization under cooling during preparation which leads to the accumulation of the drug molecules in the outer layers of the nanogels and their relatively rapid escape upon contact with the release medium. On the other hand, increasing the concentration of P188 increased MRT; which may be rationalized by the increase of PS, hence decrease of the surface area contacting the release medium resulting in slowing down of the drug release. The combination between X1 and X2 had a synergistic effect.

The 3D surface response plot representing Y5 is shown in Figure 1B.

The interactions between factors in all the equations displayed above were of second-order, and higher-order interactions equations were of less significance than the presented model; this finding runs in to agree with that of Taylor’s series assumption [40].

### 2.2. Characterization of the Optimum Formula

Based on the results displayed above, and after taking into consideration the significant models representing the PS and the MRT (Table 2 and Table 3); HNCN7 was selected as the optimum formulation with the most suitable properties and most extended release; hence it was subjected to further investigations.

#### 2.2.1. Morphological Examination

**Figure 3:**
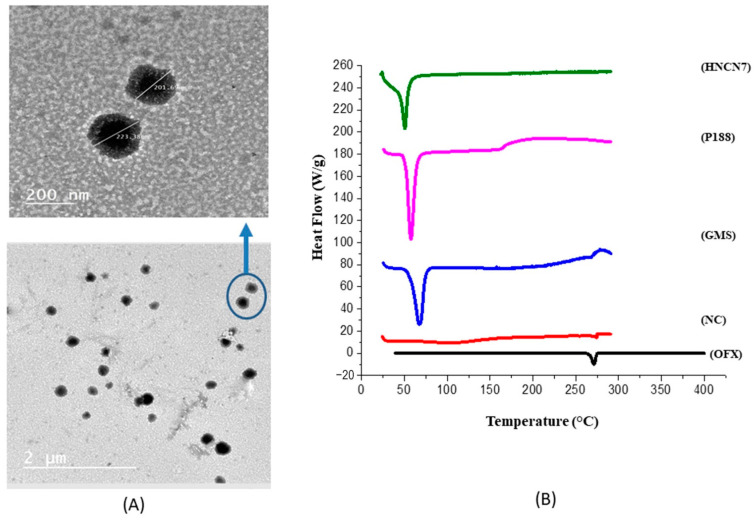
(**A**) Transmission electron micrographs (TEM) of the optimum formula HNCN7, (**B**) DSC thermograms of ofloxacin (OFX), nanocellulose crystals (NC), glyceryl monostearate (GMS), poloxamer 188 (P188) and the optimum formula HNCN7.

The morphological properties of the optimized nanogels were detected using the TEM. HNCN7 photograph showed well-separated uniform spherical nanogels with a distinct core-shell structure as seen in Figure 3A. The shell may be due to the accumulation of nanocellulose molecules at the interface, as discussed above. The nanogels size was correlated with the size detected by the Zetasizer.

#### 2.2.2. Differential Scanning Calorimetry

Figure 3B illustrates the thermal behavior of the pure OFX, GMS, NC, poloxamer 188 compared with that of HNCN7 from 25 °C to 300 °C. The sharp endothermic peak at 276.92 °C; which is recognized to OFX melting temperature indicates its crystallinity [41]. Likewise, the DSC thermograms of GMS and P188 showed endothermic peaks at 62.4 °C and 59.9 °C, respectively, which correspond to their melting points [42]. A shoulder appearing at 275 °C in the NC thermogram indicates the glass-rubber transition temperature (*Tg*) that confirms the high thermal stability of NC [43]. This shoulder is not present in the HNCN7 thermogram, and this may be due to the incorporation of NC molecules within the nanogels as NC can be adsorbed and arranged at the oil/water interface, as discussed above. Also, the HNCN7 thermogram showed the absence of the distinctive peak of OFX, assuring that OFX was wholly dispersed and integrated within the created nanogels and was transformed from a crystalline to an amorphous form.

#### 2.2.3. pH Measurement

The pH measured for HNCN7 was 6.12 ± 0.025, this value is well-suited with the skin pH between 5.0–7.0 [44], consequently favoring its safe dermal application.

### 2.3. Microbiological Studies

#### 2.3.1. Sensitivity Test and Measurement of the MIC

**Figure 4:**
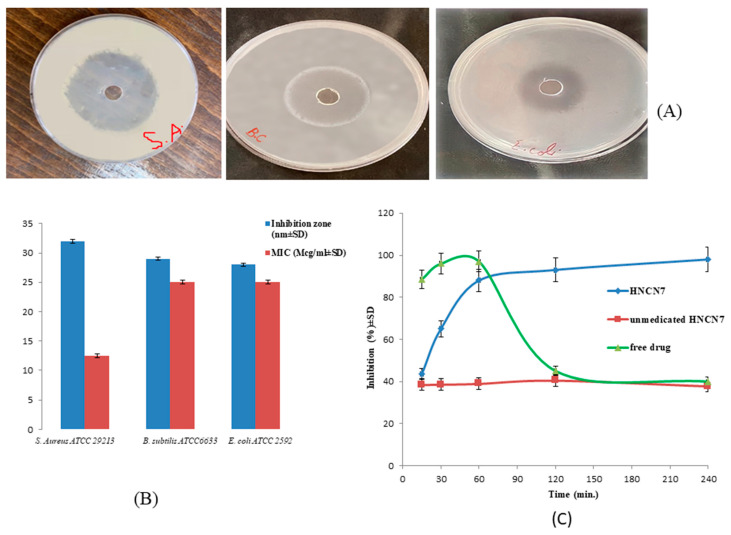
(**A**) The inhibition zones produced by OFX-loaded HNCN7 against *Escherichia coli, Bacillus subtilis, and Staphylococcus aureus* (**B**) The inhibition zones and the MICs produced by OFX-loaded HNCN7 against *Escherichia coli*, *Bacillus subtilis*, and *Staphylococcus aureus and* (**C**) In vitro time-dependent antibacterial assay for HNCN7, unmedicated HNCN7 (blank) and the free drug using *S. aureus* as test organism.

The diameter of the inhibition zones was measured to determine its inhibitory effect on the progression of the investigated bacterial strains. OFX had an antibacterial effect against all the tested microorganisms, as shown in the Figure 4A. The inhibition zone widths generated after 24 h of incubation with the studied bacteria are shown in Figure 4B, the results revealed the highest inhibitory effect against *S. aureus ATCC29213* (inhibition diameter = 32 ± 0.3 mm and MIC = 12.5 μg/mL). Consequently, *S. aureus* culture was employed to continue the antibacterial assay study.

#### 2.3.2. In-Vitro Time-Dependent Antibacterial Assay

Figure 4C shows the bacterial growth inhibition at different time intervals for the drug-loaded selected hybridized nanogels (HNCN7) compared to the blank formula (unmedicated HNCN7) and the free drug suspension. The drug-loaded HNCN7 showed a gradual increase in the degree of growth inhibition till reaching plateau after about 1 h, while the free drug showed a higher inhibition at the beginning, which then sharply decreased after 1 h. This can be explained by the continuous release of an amount of the drug sufficient to inhibit the bacterial growth over 4 h in the case of the drug-loaded hybridized nanogels, while the drug release exhibited a comparatively faster in case of the free drug suspension till consuming all the drug amount and failing to keep the antibacterial effect.

On the other side, the unmedicated formula did not show any antibacterial effect over the tested period.

### 2.4. Biological In Vitro Assay

#### 2.4.1. Cytotoxicity Study

**Figure 5:**
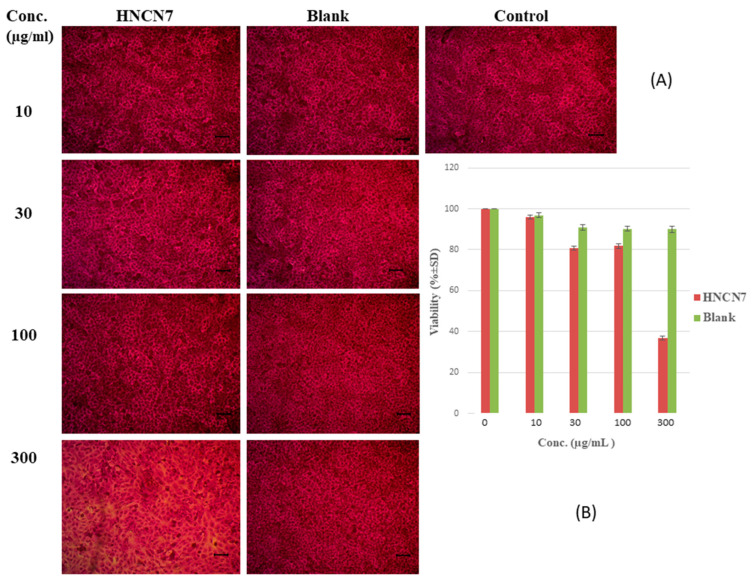
(**A**) Optical microscopy images of cells growth in presence of different concentrations of HNCN7 and the unmedicated HNCN7 (blank) by SRB assay and (**B**) Cell viability of human skin fibroblast cultured in presence of different concentrations of HNCN7 and the unmedicated HNCN7 (blank) (scale bar = 1 µm).

The SRB assay was performed to study the cytocompatibility and cell viability of the formulated HNCN7 and its corresponding blank formulation on human skin fibroblast (HSF) for 72 h. The optical microscopy images illustrated in Figure 5A show the morphology and the cell growth distribution compared to control, proving the safety of the tested OFX-loaded (HNCN7) as well as the blank formula (unmedicated HNCN7) at different concentrations. Figure 5B shows the cell viability diagram for the tested samples after 72 h. The findings showed augmented cell viability for both the medicated and the unmedicated formula at all tested concentrations up to 100 μg/mL. At a concentration of 100 μg/mL, the cell viability was 81.87 ± 0.12% and 90.28 ± 0.38% for HNCN7 and the unmedicated formulation, respectively. However, above this concentration, a decline in the cell viability was remarked in the case of HNCN7. Therefore, a concentration of 100 μg/mL was used in the following experiment.

#### 2.4.2. Experimental Scratch Assay

This assay is considered as the most uncomplicated and costless test used to examine the in vitro wound healing analysis based on cell migration [45].

**Figure 6:**
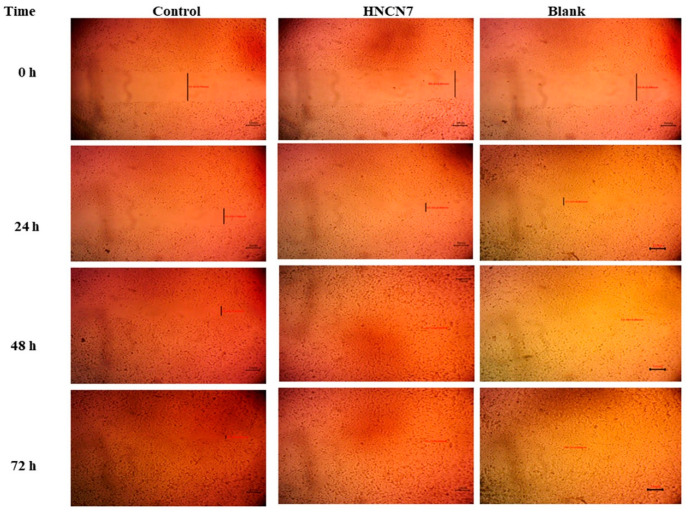
Skin repair micrographs after application of HNCN7 and the unmedicated HNCN7 (blank) for 72 h. (Scale bar = 1 mm).

**Figure 7:**
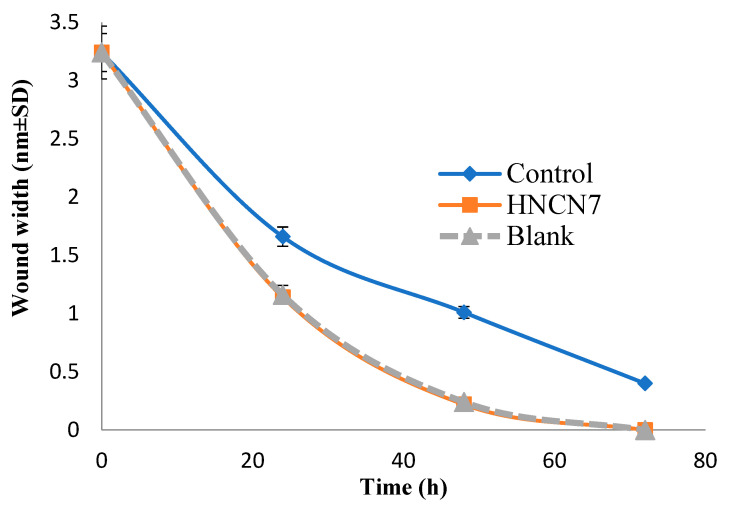
Scratch width area after application of HNCN7 and the unmedicated HNCN7 (blank).

After performing the skin scratch, the cells migration was examined from side to side for the artificial wound for 72 h. Figure 6 shows the cell migration to the scratched area for HNCN7 and its corresponding blank compared to the control. The scratch width was measured as the average distance between the edges of the scratches; as cell migration is induced, the scratch width decreases. The scratch width decreased by time in all cases, as shown in Figure 6 and Figure 7 as an indication of healing. However, HNCN7 and blank exhibited more rapid healing properties and higher gap closure efficiency within 48 h (0.22 and 0.24 mm, resp.) compared to the control (1.01 mm) (*p* < 0.5), the latter was not able to achieve a complete scratch closure even after 72 h. This study proves the curing high capability of the designed formula, which can be attributed to the intrinsic properties of its ingredients. Previous literature has proved the skin protective effect of lipid-based nanostructured preparations [22,23]. On the other hand, the cell proliferative and regenerative effects of nanocellulose are well-documented [13,17].

## 3. Conclusions

Nanocellulose crystals, in combination with a lipid, were successfully used to prepare hybridized nanogels loaded with Ofloxacin to promote skin repair while inhibiting bacterial infection. A computational method based on regression analysis was employed for the evaluation, and the results showed a good correlation between the actual and predicted values. The optimized nanogel with a high drug encapsulation efficiency and an extended drug release profile was tested biologically. It was cytocompatible and attained a prolonged inhibition of bacterial growth compared to the free drug. Moreover, it showed a faster and more efficient in vitro skin healing compared to the control. These findings emphasize the notable role of nanocellulose in regenerative medicine. Further clinical studies are necessary to prove the efficacy of this valuable biomaterial.

## 4. Materials and Methods

### 4.1. Materials

Ofloxacin (OFX) was a kind gift from Rameda Pharmaceuticals, 6th of October, Giza, Egypt. Kolliphor P188 (P188) (triblock copolymer of polyoxyethylene-polyoxypropylene, M.W. 162.23), glyceryl monostearte (GMS) (1-Stearoyl-*rac*-glycerol, M.W. 358.56), and cellulose membrane (molecular weight cut-off 12,000–14,000 Da) were purchased from Sigma Aldrich, St. Louis, MO, USA. Spray-dried Nanocellulose crystals (NC) isolated from wood was purchased from Celluforce, Quebec, Canada. HSF (Human Skin Fibroblast) cell line was purchased from Nawah Scientific Inc., (Mokatam, Cairo, Egypt). All other reagents were purchased from El-Nasr Company for Pharmaceutical Chemicals, Cairo, Egypt.

### 4.2. Methods

#### 4.2.1. Design of Experiments (DOE) and Regression Analysis

A 2^3^ full factorial experimental design (Table 3) was followed to explore the impact of the selected variables on the characteristics of the prepared formulations. The chosen independent factors were lipid concentration (X1), surfactant concentration (X2) and NC concentration (X3). The measured responses were particle size (PS), polydispersity index (PDI), Zeta potential (ZP), encapsulation efficiency (EE) and mean release time (MRT), presented as Y1, Y2, Y3, Y4 and Y5, respectively. The software (MS-Excel) was employed for the statistical analysis of the factorial design based on multiple linear regression analysis [46,47,48,49]. Statistical validation of the generated equation utilizing ANOVA was performed.

Y = b0 + ∑ biXi + ∑ bijXiXj

where Y represents the measured response, b0 represents the intercept which is the arithmetic average of the quantitative outcomes of experimental runs; bi is the coefficient calculated from the practical experimental values of Y. At 10% confidence level, the values of the correlation coefficient are contemplated statistically significant. Xi represents the main effect and is the result of changing a variable from its low to high levels. XiXj represents the interaction between the individual variables. The most significant model for each response is selected. Regression equations are employed to deduce the conclusions based on the magnitude and a mathematical sign of the coefficients. Positive signs indicate that the effects favor the optimization, whereas negative ones indicate the contrary. For interactions, the positive sign indicates synergism, and the negative sign indicates antagonism.

#### 4.2.2. Preparation of Ofloxacin-Loaded Hybridized NC/Lipid Nanogels

OFX-loaded hybridized NC/lipid nanogels (OFX-HNCNs) were prepared by the hot homogenization method followed by ultrasonication [50]. GMS (2% or 4% *w*/*v*) was melted and OFX (0.8% *w*/*v*) was dispersed in the melted lipid, and then this lipid phase was poured on the aqueous phase containing P188 (5% or 10% *w*/*v*) and NC (1% or 2% *w*/*v*) under homogenization for 5 min at 22,000 rpm (Heidolph Homogenizer, Schwabach, Germany). The obtained preparation was sonicated for 30 min and left to cool down at room temperature for lipid phase recrystallization [51], and finally stored at room temperature for further investigations.

#### 4.2.3. Evaluation of Ofloxacin-Loaded Hybridized NC/Lipid Nanogels

##### Encapsulation Efficiency (EE)

The encapsulation efficiency of OFX within the prepared formulations was calculated indirectly by supernatant collection after centrifuging the formulations using a cooling centrifuge (Union 32R, Gimpo, Korea) at 9000 rpm for 90 min. The concentration of the free OFX in the supernatant was measured spectrophotometrically using a UV-visible Spectrophotometer (Shimadzu UV spectrophotometer, 2401/PC, Tokyo, Japan), the EE% was computed as follows [52,53]:

$$\mathrm{EE}\left(\%\right)=\frac{\mathrm{Initial}\;\mathrm{OFX}\;\mathrm{concentration}\;-\;\mathrm{Free}\;\mathrm{OFX}\;\mathrm{concentration}}{\mathrm{Initial}\;\mathrm{OFX}\;\mathrm{concentration}}\times 100$$

##### Estimation of Particle Size, Zeta Potential, and Polydispersity Index Analysis

A Zetasizer (Malvern Instrument, Worcestershire, UK) was used to estimate the average particle size (PS), the surface charges or Zeta potential (ZP) and the polydispersity index (PDI) values of the prepared nanogels. Before analysis, the dispersion was diluted tenfold with deionized water. Value represents an average of three measurements at room temperature, at a 90 °C fixed angle.

##### In-Vitro Release Studies

The dialysis bag method, which is commonly used for the evaluation of drug release from nano-carriers, was utilized for the in vitro release of OFX from the nano-formulations [54]. The cellulose membrane was filled with a specific volume of OFX-loaded HNCN (1 mL), and the bag’s end was securely closed. The bags were submerged in 100 mL of phosphate buffered solution (pH 7.4) denoting the receptor compartment, agitated at a rate of 100 rpm and held at 37 ± 0.5 °C [55,56]. Samples are withdrawn at preset intervals of time (0.5, 1, 2, 3, 4, and 6 h). The OFX percentage released was deliberated spectrophotometrically (at 288 nm). By replenishing the withdrawn samples with fresh ones, the release medium was kept at a constant volume. Results represented the mean values of three replicates ± S.D. In order to compare the drug release behavior of the tested formulae, the mean release time (MRT) was computed using the following equation:

$$\mathrm{MRT}=\frac{\sum_{i=1\;}^{n}t_{\mathrm{mid}}\Delta M}{\sum_{i=1\;}^{n}\Delta M}$$

in which i represents the number of the sample, n stands for the sample times number, t_mid_ presents the midpoint amongst times ti and ti − 1, whereas ΔM represents the drug amount released (mg) between the latter times. The MRT is an approach that translates the release profile into a single value. Increased MRT means a slower drug release [57].

#### 4.2.4. Characterization of the Optimized HNCN

Based on the measured significant responses, and with target criteria of minimizing particle size and maximizing MRT, the optimum HNCN formulation was selected and subjected to further investigations using the desirability function.

##### Differential Scanning Calorimetry (DSC)

Using the Differential Scanning Calorimetry DSC131 evo (SETARAM Inc., Caluire, France), the thermal characterization of the selected HNCN and its constituent components was investigated. The instrument was calibrated using the standards (Mercury, Indium, Tin, Lead, Zinc, and Aluminum). Nitrogen and helium were employed as purging gases. The test was programmed including the heating zone with a temperature ranging from 25 °C to 300 °C and a heating rate 10 °C per minute. The samples were weighed in a 120 uL Aluminum crucible before being introduced into the DSC. The obtained thermograms were assessed for peaks change or the development of new peaks.

##### pH Measurement

One ml of the selected HNCN was diluted in 10 mL of distilled water and measured at 25 °C using a digital Jenway pH meter (Bibby Scientific Limited, Staffordshire, UK) following standardization of the instrument utilizing pH 7.0 and pH 10.0 buffered solution. All measurements were carried out in triplicates.

##### Morphological Examination

The morphological characteristics of the optimized nanogels were examined using transmission electron microscope (TEM) (JEM-1230, JEOL, Akishima, Japan). The chosen formula diluted using distilled water (1:10) and applied on carbon film-coated copper grids. Samples were stained negatively using 2% (*w*/*w*) phosphotungstic acid solution.

#### 4.2.5. Microbiological Studies

##### Antimicrobial Sensitivity Test

The agar well diffusion method was used to test the sensitivity of OFX [58]. About 100 µL of suspension containing 1 × 10^6^ CFU/mL of three pathogenic bacterial strains: *Bacillus subtilis ATCC6633* and *Staphylococcus aureus ATCC 29213*, as Gram + ve bacteria, and *Escherichia coli ATCC 2592* as Gram -ve bacteria. The bacterial suspensions were spread on nutrient agar after the media had cooled and solidified; wells (10 mm in diameter) were formed in the solidified agar and loaded with 100 μL of tested sample. Then, all plates were subjected to incubation for 24 h at 37 °C. The antimicrobial activity was assessed after the incubation period by determining the inhibition zone (mm) and compared against the standard.

##### Minimal Inhibitory Concentration (MIC) Measurement

The bacteriostatic activity of the selected OFX was assessed using the two-fold serial dilution method [59]. Using the suitable nutrient broth, the serial dilutions of OFX were prepared. The solutions’ final concentrations were 100, 50, 25, and 12.5 µg/mL. After that, the tubes were infected with bacterial broth (100 µL) inclosing 1 × 10^6^ CFU/mL of the chosen test microorganism. The tubes were incubated for 24 h at 37 °C. The lowest concentration showing no bacterial growth was considered as the minimum inhibitory concentration (MIC).

##### In-Vitro Time-Dependent Antibacterial Assay

This assay was done based on a previously described method to assess the antimicrobial activity over a certain period of time [60,61], with some modifications to suit the current formulation. The antibacterial effect of the drug-loaded formula (HNCN7) and the corresponding blank one (unmedicated HNCN7) was compared to the free drug aqueous suspension containing an equivalent amount of the drug. The experiment was run following the same procedure of the in vitro drug release experiment to test the antibacterial activity of the withdrawn samples at predetermined timings of 15, 30, 60, 120, and 240 min. The withdrawn samples were added to the tubes previously inoculated with 100 μL of bacterial broth containing 1 × 10^6^ CFU/mL of the chosen test microorganism (*Staphylococcus aureus ATCC 29213*). The inoculated tubes were incubated for 24 h at 37 °C. Using a UV spectrophotometer, the inhibition was determined turbidimetrically at λmax 600 nm (Jenway Model 6315, UK). All the experiments were performed in triplicates, and the results were calculated using the arithmetic mean.

#### 4.2.6. Biological In-Vitro Assay

##### Cytotoxicity Study

Human skin fibroblast (HSF) cell line was utilized for testing the cytotoxic activity of the selected HNCN and its corresponding unmedicated formulation (blank). In a humidified, 5% (*v*/*v*) CO_2_ atmosphere, cells were kept in Dulbecco’s modified Eagle’s medium (DMEM) enhanced with streptomycin (100 mg/mL), penicillin (100 units/mL), and 10% heat-inactivated fetal bovine serum.

The Sulforhodamine B (SRB) assay was chosen to study the cell viability [62]. In 96 well plates, aliquots of 100 μL cell suspension (5 × 10^3^ cells) were cultured and incubated for 24 h in complete media. Another aliquot of 100 μL media containing the selected drug-loaded formula (HNCN7) and the unmedicated HNCN7 (blank) at different concentrations were treated with the cells. After 72 h of exposure, fixed of the cells was done by replacing the media with 150 µL of 10% trichloroacetic acid (TCA) followed by incubation at 4 °C for 1 h.

Distilled water was used for cell washing (five times) after removing the TCA solution.

Followed by addition of aliquots of 70 µL of 0.4% *w*/*v* SRB solution and incubated in dark for 10 min at room temperature. After which 1% acetic acid was used for plates washing for three times and air-dried for 24 hr. BMG LABTECH^®^-FLUO star Omega microplate reader (Ortenberg, Germany) was utilized for measuring the absorbance at 540 nm using a after 150 µL of Tris base solution (10 mM) was added to dissolve protein-bound SRB stain.

##### Experimental Scratch Assay

HSF cell line was grown in DMEM enhanced with streptomycin (100 mg/mL), penicillin (100 units/mL), and 10% heat-inactivated fetal bovine serum. The medium was kept in a humidified atmosphere by 5% (*v*/*v*) CO_2_ at 37 °C.

Cells were plated onto a coated 9-well plate at density of 3 × 10^5^/well for scratch wound assay and cultured overnight at 37 °C in 5% FBS-DMEM and 5% CO_2_. On the following day, the confluent monolayer was horizontally scratched followed by washing the plate extensively with PBS, control wells were replaced with the fresh medium while HNCN7 wells and blank wells were treated with fresh media inclosing the drug-loaded HNCN7 and the unmedicated HNCN7 (100 μg/mL), respectively [63,64,65]. An inverted microscope was used for taking mages at the indicated time intervals (0, 24, 48 and 72 h). In the intervening time, the plate was incubated in 5% CO_2_ at 37 °C. The acquired images were analyzed by MII Image View software version 3.7. Wound widths were calculated as the average distance between the edges of the scratches. The results are displayed as mean ± standard deviation.

##### Statistical Analysis

SPSS^®^ software (SPSS Inc., Chicago, IL, USA) was used to analyze the data. The one-way analysis of variance (ANOVA) followed by the least significant difference (LSD) post-hoc test were used to determine the significance of differences. Statistical significance was defined as a *p* value of less than 0.05.

## Figures and Tables

**Table 1:** Particle size (PS), size distribution (PDI), Zeta potential (ZP), encapsulation efficiency (EE), mean release time (MRT) of the prepared HNCNs and Model summary statistics for the influence of formulations variables on the measured responses *.

Formulae	PS ± SD (nm)	PDI ± SD	ZP ± SD	EE (%) ± SD	MRT ± SD (h)
**HNCN1**	244.9 ± 6.45	0.6 ± 0.03	−26.6 ± 1.22	95.80 ± 2.01	0.89 ± 0.02
**HNCN2**	265.1 ± 10.01	0.4 ± 0.05	−26.1 ± 0.03	97.13 ± 1.23	0.54 ± 0.03
**HNCN3**	254.2 ± 8.22	0.4 ± 0.01	−27.3 ± 1.25	97.73 ± 1.36	0.94 ± 0.10
**HNCN4**	268.3 ± 11.32	0.6 ± 0.02	−22.3 ± 1.01	96.56 ± 2.30	0.74 ± 0.06
**HNCN5**	173.5 ± 11.01	0.4 ± 0.02	−27.9 ± 0.50	97.05 ± 2.01	0.83 ± 0.07
**HNCN6**	190.1 ± 5.50	0.5 ± 0.01	−29.6 ± 0.84	96.56 ± 1.22	0.46 ± 0.03
**HNCN7**	200.2 ± 6.74	0.5 ± 0.01	−26.4 ± 0.50	97.53 ± 1.56	0.93 ± 0.11
**HNCN8**	214.5 ± 5.90	0.6 ± 0.03	−26.5 ± 0.77	97.86 ± 2.02	0.73 ± 0.07
**Best fitting equation**	226.25 + 8.15X1 + 7.88X2 − 31.73X3 − 1.03X1X2 − 0.38X1X3 + 4.85X2X3	0.53 + 0.028X1 + 0.022X2 + 0.007X3 + 0.065X1X2 + 0.025X1X3 − 0.045X2X3	−26.59 + 0.46X1 + 0.96X2 − 1.01X3 + 0.76X1X2 − 0.91X1X3 + 0.19X2X3	97.02 + 0.0001X1 + 0.39X2 + 0.22X3 − 0.21X1X2 − 0.04X1X3 + 0.05X2X3	0.76 − 0.14X1 + 0.08X2 − 0.02X3 + 0.04X1X2 − 0.001X1X3 + 0.01X2X3
**Significance**	Significant	Not Significant	Not Significant	Not Significant	Significant
**R** **^2^**	0.9998	-	-	-	0.9998
**Adjusted R** **^2^**	0.9986	-	-	-	0.9984
**Predicted R** **^2^**	0.9876	-	-	-	0.9856
**Adeq Precision**	76.190	-	-	-	71.813
**Reduced model**	226.25 + 8.15X1 + 7.88X2 − 31.73X3 + 4.85X2X3	-	-	-	0.76 − 0.14X1 + 0.08X2 + 0.01X1X2

* Values represent mean ± SD.

**Table 2:** Summary of results of the regression analysis of the significant models *.

Terms	Y1 (PS)		Y5 (MRT)	
	Effect	*p* Value for Coefficients of Factors	Effect	*p* Value for Coefficients of Factors
b0	226.25	0.001	0.759	0.005
b1	8.15	0.039	−0.144	0.027
b2	7.875	0.040	0.081	0.048
b3	−31.725	0.010	−0.024	0.161
b1b2	−1.025	0.288	0.039	0.100
b1b3	−0.375	0.590	−0.001	0.874
b2b3	4.85	0.065	0.014	0.271

* *p* value was considered significant at values ≤ 0.1.

**Table 3:** Composition of the formulations based on 2^3^ full-factorial design.

Formulae	Variable Levels in Coded Form		
	X1	X2	X3
HNCN1	−1	−1	−1
HNCN2	1	−1	−1
HNCN3	−1	1	−1
HNCN4	1	1	−1
HNCN5	−1	−1	1
HNCN6	1	−1	1
HNCN7	−1	1	1
HNCN8	1	1	1
**Translation of Coded Levels in Actual Units**			
**Variable Levels**		**−1 (low)**	**1 (high)**
X1: Lipid concentration (*w/v*)		2%	4%
X2: Surfactant concentration (*w/v*)		5%	10%
X3: NC concentration (*w/v*)		1%	2%

## Data Availability

Data available within the article.
